# The Endometrial Microbiome and Its Impact on Human Conception

**DOI:** 10.3390/ijms23010485

**Published:** 2022-01-01

**Authors:** Bruno Toson, Carlos Simon, Inmaculada Moreno

**Affiliations:** 1 INCLIVA Biomedical Research Institute, Av. Menendez y Pelayo 4, 46010 Valencia, Spain; bruno.toson@igenomix.com; 2 Igenomix Foundation/INCLIVA Biomedical Research Institute, Narcis Monturiol Estarriol 11B, 46980 Paterna, Spain; 3 Department of Obstetrics and Gynecology, University of Valencia, Av. Blásco Ibáñez 15, 46010 Valencia, Spain; 4 Beth Israel Deaconess Medical Center, Harvard University, 330 Brookline Ave, Boston, MA 02215, USA; 5 Department of Obstetrics and Gynecology, Baylor College of Medicine, 1 Baylor Plaza, Houston, TX 77030, USA

**Keywords:** endometrial microbiota, microbiome, reproductive tract microbiota, uterus, reproductive outcomes, infertility, assisted reproductive technologies, human reproduction

## Abstract

Changes in the female genital tract microbiome are consistently correlated to gynecological and obstetrical pathologies, and tract dysbiosis can impact reproductive outcomes during fertility treatment. Nonetheless, a consensus regarding the physiological microbiome core inside the uterine cavity has not been reached due to a myriad of study limitations, such as sample size and experimental design variations, and the influence of endometrial bacterial communities on human reproduction remains debated. Understanding the healthy endometrial microbiota and how changes in its composition affect fertility would potentially allow personalized treatment through microbiome management during assisted reproductive therapies, ultimately leading to improvement of clinical outcomes. Here, we review current knowledge regarding the uterine microbiota and how it relates to human conception.

## 1. Introduction

### 1.1. The Human Uterus: A Non-Sterile Body Site

The human microbiome was first described as “the ecological community of commensal, symbiotic, and pathogenic microorganisms that literally share our body space” [1]. The Human Microbiome Project (HMP) [2] initiated the gathering of deep knowledge about the microbiota in different body sites, but how to define a healthy bacterial composition of the uterus remains under debate. The uterine cavity was believed to be sterile until the second half of the 20th century, when traditional microscopy and culture-based techniques were used to assess the microbiome [3,4]. With the advent of next-generation sequencing (NGS), techniques such as 16S rRNA gene sequencing enabled many studies to describe different microbial communities inside the uterine cavity, yet it is unclear where these originate. For instance, uterine colonization is hypothesized to occur from gut, oral cavity, bloodstream, and vaginal ascension [3,5], but uterine seeding could potentially occur through assisted reproductive technology (ART) procedures and placement of contraceptive devices [6,7] as well as attachment of microorganisms to human spermatozoa [8]. Regardless of its origin, research consistently demonstrates that the uterine microbiome is highly diverse and scarcely populated compared with the lower genital tract [9,10,11], but its composition remains to be fully unraveled.

### 1.2. The Composition of the Endometrial Microbiota

A number of studies describe the healthy state of the uterine microbiota in women of reproductive age, with most reporting dominance of *Lactobacillus* species. In an assessment of the upper genital tract (UGT) using endometrial swabs from 58 women undergoing hysterectomy for non-cancer indications, 95% of patients had UGT colonization with great abundance of *L. iners*, *Prevotella* spp., and *L. crispatus* [12]. Later, endometrial fluid (EF) samples from 13 fertile patients revealed an abundance of *Lactobacillus* [13]. These findings prompted a suggestion of two possible types of bacterial endometrial composition: *Lactobacillus*-dominant (LD; with *Lactobacillus* spp. abundance higher than 90%) or non-LD (NLD; *Lactobacillus* spp. abundance lower than 90%). Other genera commonly detected in endometrial fluid samples were *Bifidobacterium*, *Gardnerella*, *Prevotella*, and *Streptococcus* [13]. Endometrial tissue from 19 women of European descent with diverse medical histories exhibited 183 bacterial phylotypes, of which 15 were present in all samples. Furthermore, bacteria belonging to the *Proteobacteria* and *Bacteroidetes* phyla were the most commonly found among samples, and *Lactobacillus* spp. were predominant in six women [14]. Later, evaluation of endometrial samples from the tips of transfer catheters of 70 patients detected *Lactobacillus* in all samples. Of note, nearly half of subjects had over 90% *Lactobacillus* abundance, and 50/70 patients had over 70% *Lactobacillus* [15]. Assessment of EF microbiota in seven asymptomatic healthy volunteers later revealed that six had high abundance of *Lactobacillus*, which accounted for more than 90% of microbial composition [16]. Accordingly, 90 bacterial genera were detected in endometrial samples of 15 patients undergoing ART, with greater proportion of *Lactobacillus* and the presence of genera such as *Gardnerella*, *Prevotella*, and *Propionibacterium* in lower quantities [17]. Lastly, a recent study revealed *Lactobacillus* to be the most abundant genus in both endometrial tissue and endometrial fluid, while multiple other bacteria—namely *Anaeroccocus*, *Atopobium*, *Bifidobacterium*, and *Gardnerella*, among others—were also found in lower proportions. Further analysis enabled the establishment of bacterial networks, and *Lactobacillus* was negatively correlated with *Gardnerella*, *Bifidobacterium*, and *Atopobium* and positively associated with the commensals *Clostridium* and *Streptomyces* [18]. This group behavior was also reported in vaginal samples during the menstrual cycle and demonstrates a certain degree of dependence between some species inside the female genital tract (FGT) [19].

By contrast, some studies did not observe *Lactobacillus* predominance inside the uterine cavity. Endometrial samples in 80 Chinese women undergoing surgery for conditions other than infection were dominated by *Acinetobacter*, *Pseudomonas*, *Sphingobium*, and *Vagococcus* [9]. Accordingly, another study including 137 Chinese women undergoing gynecological surgery indicated that NLD endometrial microbiota—such as Moraxellaceae, Propionibacteriaceae, Pseudomonadaceae, and Streptococcaceae—accounted for a large fraction of the uterine microbiota [20]. In uterine samples from 25 Italian patients who underwent hysterectomy for fibroids, the endometrial microbiota was dominated by *Acinetobacter*, *Cloacibacterium*, *Comamonadaceae*, and *Pseudomonas,* while *Lactobacillus* species were rare inside the uterus [21]. In 2019, a similar study was conducted in 19 women of European descent with normal pregnancy at full-term. Analysis of endometrial biopsies revealed a common bacterial composition among subjects, suggesting that genera such as *Acinetobacter, Corynebacterium*, *Cutibacterium*, *Escherichia*, *Staphylococcus*, and *Streptococcus* could be part of an endometrial microbiota core. Results also revealed that *Lactobacillus* is present in about 20% of patients and with abundance variability below 16% [22]. More recently, an analysis of uterine microbiota from seven healthy women revealed that endometrial biopsies harbored communities of bacteria (85%), fungi (10%), viruses (5%), and archaea (0.3%); *Clostridium botulinum*, *Hydrogenophaga* sp., *Klebsiella pneumoniae*, and *Pasteurella multocida* were the most abundant microorganisms [23].

In another study, there was great dissimilarity among endometrial samples from 10 patients undergoing hysterectomy, with *Acinetobacter*, *Corynebacterium*, *Lactobacillus*, and *Staphylococcus* detected in variable abundances [24]. Of note, the study included premenopausal, perimenopausal, and postmenopausal patients, a design that could greatly impact results and study comparison because of the natural changes occurring in microbiota across the lifespan [25]. Interestingly, endometrial microbiota from 34 women of European descent undergoing ART treatment showed six possible biomarker species for the endometrium—namely *Kocuria dechangensis*, *Sphingomonas paucimobilis*, *Stenotrophomonas maltophilia*, *Agrobacterium tumefaciens*, *Delftia tsuruhatensis*, and *Cutibacterium acnes*—together with an almost total absence of *Lactobacillus* [26]. Of note, some of these species are found mainly in soil or in water, indicating a source of contamination during the analysis. These results demonstrate the importance of experimental controls and adequate handling of samples and reagents since environmental contamination can greatly impact study quality.

## 2. Variations in Endometrial Microbiota

There are significant fluctuations in bacterial communities in the FGT. These shifts correlate with parameters such as age, hormonal changes, ethnicity, and intrauterine devices [9,17,19,25,27]. Although most data come from vaginal samples given the site’s easy accessibility and lower risk of sample contamination, there is increasing knowledge about changes in the endometrial microbiome. For instance, sexual activities appear to influence the microbiota taxa; in one study, a greater diversity of bacterial taxa occurred among virgo intacta subjects whose samples were dominated by obligate anaerobes *Fusobacterium* and *Jonquetella*, while *Lactobacillus* spp. numbers were decreased [28]. Furthermore, there is a significant impact of age in bacterial communities inside the uterus. Microbial diversity within samples (alpha diversity) decreases with advancing age, while interindividual similarity (beta diversity) is higher among women ages 20 years and younger, suggesting that dissimilarities among patients accumulate with age [25]. Older age, number of abortions, and vaginal delivery may reduce the differences between endometrial and vaginal microbiomes via perturbation of the closed uterine environment through cervical incompetence, which ultimately suggests that alterations in the vaginal microbiome could directly affect bacterial communities in the endometrium in certain circumstances [25]. This theory is corroborated by findings that the majority of patients with an NLD endometrial microbiota also had an NLD vaginal microbiota, and all patients with dysbiotic vaginal communities had an NLD endometrial status [16]. Notably, oral contraceptives and intrauterine devices—such as copper and levonorgestrel-releasing systems—might also lead to changes in the vaginal microbiota [27,29] and could potentially impact the endometrial microbiome.

The endometrial microbiome is significantly impacted by hormonal changes. For example, exogenous progestin significantly alters the endometrial microbiota, including by decreasing diversity in *Lactobacillus* spp. phylotypes [28]. Later findings demonstrated that controlled ovarian stimulation and exogenous progesterone lead to significant changes and a greater diversity index in both the vaginal and endometrial microbiota. Additionally, abundance of bacteria such as *Atopobium* and *Prevotella* increased after treatment, while the proportion of *Lactobacillus* slightly decreased [17]. Of note, naturally occurring hormonal swings during the menstrual cycle correlate to the instability of microbial communities, and regular replacement of bacterial species throughout the cycle occurs in vaginal samples [19]. Significant changes also occur in the endometrial microbiome, and increased numbers of *Prevotella* spp. and *Sneathia* spp. may be hallmarks of the proliferative and secretory phases, respectively [28]. *Propionibacterium*, *Pseudomonas,* and *Sphingobium* species vary in abundance at different times across the menstrual cycle, with upregulated bacterial proliferation during the proliferative phase and a higher abundance of peptidoglycan synthesis and aminoacyl-tRNA synthesis [9]. Moreover, the proportion of *Lactobacillus* was reportedly low after menstruation but gradually increased during follicular development and reached its peak during the luteal phase [30]. Interestingly, the abundance of not only bacteria, but also viruses and archaea, differ significantly between mid-secretory and proliferative phases [23]. In addition, the uterine microbiome appears relatively stable during acquisition of endometrial receptivity, as sequencing did not reveal significant differences in bacterial communities comparing endometrial samples collected 2 and 7 days after the luteinizing hormone (LH) peak [13].

Natural swings could be considered before different treatment approaches (e.g., administration of antibiotics or probiotics) are chosen, as a suboptimal microbial taxonomic composition could potentially transition to a healthy and eubiotic state in a short period of time. It is also important to consider that increasing maternal age is one of the main reasons why patients pursue in vitro fertilization (IVF) treatment. Thus, although some microbiome changes may be cyclic, postponing embryo transfers because of dysbiotic endometrial states without any practical approaches for microbiome management may not be ideal in some cases. 

## 3. Virulence Mechanisms of a Dysbiotic Endometrial Microbiome

Several different mechanisms are proposed to explain how changes of bacterial communities inside the uterine cavity could lead to infertility and other obstetric conditions. *Lactobacilli* play an important role in maintenance of vaginal bacterial communities through production of bacteriocins, hydrogen peroxide, and lactic acid, which decreases the vaginal pH and impairs growth of pathogenic bacteria. Furthermore, competitive adhesion to the vaginal epithelium and immune response modulation may also maintain a healthy genital tract by creating a hostile environment for pathogenic bacteria [31,32]. Nonetheless, distinct bacterial communities are able to thrive through virulence mechanisms such as mucin degradation, biofilm formation, and antimicrobial resistance, leading to dysbiotic states [33]. Although there is no consensus on the endometrial microbiota, commensal bacteria inside the uterine cavity could also help maintain an eubiotic state. 

Some hypotheses speculate that a decrease in the number of *Lactobacilli* would lead to a significant increase in the FGT pH, which could impact embryo attachment ability and allow proliferation of bacterial communities that would not have proper growth conditions in a normal pH range [34]. In fact, gynecological conditions such as bacterial vaginosis are related to both elevated vaginal pH and decreased *Lactobacillus* colonies [35,36]. However, assessment of pH value as a potential predictor of endometrial microbiota in 14 endometrial fluid samples revealed a wide range of pH values regardless of the bacterial composition [13], suggesting that the abundance of *Lactobacilli* alone is not enough to change the uterine pH. Of note, similar results were observed in the vaginal microbiome, as no association between vaginal pH and *Lactobacillus* abundance was found [37]. These results indicate that other changes in the endometrium besides pH are related to gynecological and obstetrical pathologies and should be deeply investigated.

Studies of the FGT metabolome and proteome revealed significant differences between healthy and infertile patients. Protein expression patterns in EF samples from 110 patients undergoing fertility treatment revealed enrichment of processes related to immune response, inflammation, and cell–cell adhesion in non-pregnant women [38]. Assessment of 11 endometrial fluid (EF) samples detected naturally occurring peptides with antimicrobial activity [39], which are known for their protection against a myriad of pathogens, including bacteria, viruses, and fungi and are present in several parts of the FGT [40,41]. Considering that microbiota changes are related to several gynecological and obstetrical disorders, these peptides could directly impact bacterial community balance and may be major players in establishing a healthy environment for embryo implantation and development [40]. Furthermore, patients with repeated implantation failure (RIF) had significantly different metabolites in vaginal samples, and some of these compounds were either positively or negatively correlated to *Lactobacillus* abundance [42]. Variations in microbial taxa impact the vaginal metabolome, although certain fluctuations in microbiota composition do not lead to metabolite differences because of functional redundancy among bacterial species [19]. Thus, bacterial taxon imbalances could also impact metabolic composition of the uterine cavity, enabling growth of pathogenic bacteria in this dysregulated environment. Interestingly, metabolome analysis of vaginal samples was demonstrated to successfully predict microbiota composition through abundance of metabolites such as thiomalic and lignoceric acids [43], and similar techniques could potentially be a substitute for endometrial biopsies. Although metabolome analysis might not allow taxonomic resolution at the species level, this kind of assessment would be less invasive and deserves thorough consideration. Nonetheless, it is important to state that such variations in metabolomes could result in or from fluctuations in bacterial community composition, and further studies are needed to elucidate the mechanisms involved in these processes.

Inflammatory responses to bacterial modifications might also be related to infertility, as proinflammatory microenvironments occur in different gynecological and obstetrical diseases. For instance, there is increased expression of immunoglobulins IgM, IgA, and IgG in patients with chronic endometritis (CE) and RIF [44], and significantly higher levels of the proinflammatory cytokines interleukin 6 (IL-6), proIL-1β, and IL-1β occur among patients with endometriosis [45,46]. A separate study reported corroborating results and demonstrated higher risks for inflammatory bowel disease in patients with endometriosis [47]. Additionally, strict modulation of the immune response through regulatory T (Treg) cells and cytokines plays important roles in immune tolerance and maintenance of pregnancy [48,49,50,51]. Cytokines and other immune modulators are also related to embryo implantation, as disturbance of IL-11, leukemia inhibitory factor, and transforming growth factor led to implantation failure and abnormal placental formation [52]. Of note, expression of cytokines is greatly modulated by Toll-like receptors (TLRs), which are highly expressed in the reproductive tract and recognize pathogen-associated molecular patterns, such as lipopolysaccharide and peptidoglycan [53]. The impact of TLRs in pregnancy outcomes is reviewed elsewhere [54], and it is feasible to speculate that fluctuations in bacterial communities induce an immune response from the host inside the genital tract, increasing production of proinflammatory cytokines that could impair embryo implantation and worsen clinical outcomes during ART cycles.

Given that little is known about the interaction between the endometrial microbiome and the host’s immune response, further assessment is needed to clarify how specific bacterial species can modulate inflammation. Nonetheless, the anti-inflammatory role of *Lactobacillus* spp. in the vaginal microenvironment has been reviewed elsewhere [32], and we can speculate that *Lactobacilli* could also contribute to uterine homeostasis by inducing secretion of anti-inflammatory cytokines such as IL-1RA and producing antimicrobial peptides. Nevertheless, it is important to consider that immuno-dysregulated states might not be a consequence of microbiome changes but rather the dysbiotic environment in which distinct bacteria can be found. Of note, successful treatment with antibiotics alone did not lead to better outcomes in patients with RIF [55], which supports the idea that increased inflammatory signaling impairs reproductive outcomes, and treatments focused on microbiota modulation might not be highly effective if implemented alone.

In general, these results demonstrate that changes in microbiota alone might not fully account for decreased reproductive outcomes, but host responses to dysbiotic bacterial states might also play central roles in driving infertility. Thus, in-depth understanding of microbiota–host interaction mechanisms may provide valuable information on how fluctuations in endometrial microbiota are related to ART treatment success, and future studies should assess this topic further.

## 4. Microbiota Alterations in Infertility

Increasing evidence demonstrates the impact of variations in the FGT microbiota on overall female health [56]. For instance, several microorganisms related to bacterial vaginosis (BV)—such as *Gardnerella vaginalis*, *Atopobium vaginae*, and *Prevotella bivia*—are associated with the occurrence of chronic endometritis and pelvic inflammatory disease [57,58,59]. Thus, considering the impact of such conditions on fertility, further understanding of how swings in endometrial microbiota are associated with these pathologies may also provide important insights into new treatments through microbiota management.

### 4.1. Endometriosis

Analysis of endometrial samples from 73 women with endometriosis and 55 controls revealed significantly greater colony-forming units (CFU) of *Enterococcus*, *Gardnerella*, and *Streptococcus* than in the control group [60]. Further investigation in 64 women demonstrated that patients with endometriosis have lower numbers of Lactobacillacae in the uterine cavity and increased abundance of Moraxellaceae and Streptococcaceae [61]. An assessment of 21 endometrial samples revealed lower diversity in the control group and enrichment of several bacteria—such as the Actinobacteria phylum, Oxalobacteraceae, and Streptococcaceae families—in samples from patients with endometriosis [62]. Yet, a separate study found no significant changes in microbial composition in patients with endometriosis whose samples were comparable to the control group and were composed mainly of the genera *Gardnerella*, *Lactobacillus*, *Prevotella*, and *Streptococcus*. Further assessment revealed that deep endometriotic lesions presented distinct microbiota, with higher numbers of *Alishewanella*, *Enterococcus*, and *Pseudomonas* [63], and a significant shift in microbiota composition in a patient with stage III endometriosis has also been reported [64]. Among 26 patients with endometriosis and 11 controls, although *Lactobacillus* presence was homogeneous through the lower genital tract in the control group, endometriosis patients had a decrease of *Lactobacillus* abundance in the endometrium compared with cervical mucus. Moreover, endometriosis patients had a distinct uterine bacterial composition, with enrichment of samples dominated by a mixture of *Acinetobacter*, *Pseudomonas*, and *Vagococcus* in this group [65]. In fact, a recent systematic review suggested that increased abundance of bacteria such as Enterobacteriaceae, *Escherichia coli*, Proteobacteria, and *Streptococcus* in different sites appears to be associated with endometriosis [66]. Further studies regarding microbiota in patients with endometriosis could provide valuable insights into new diagnostic and prognostic tools.

### 4.2. Chronic Endometritis

Findings in 2008 revealed significantly higher positive endometrial cultures in women with CE than in the control group, with *Ureaplasma urealyticum* detected in 10% of cases [67]. Subsequently, endometrial biopsies and fluid from 130 infertile patients exhibited lower abundance of *Lactobacillus* in endometrial samples from the CE group than in samples from the non-CE group, along with higher numbers of *Anaerococcus*, *Bifidobacterium*, *Dialister*, *Gardnerella,* and *Prevotella* [68]. Similarly, a study including 60 patients undergoing IVF treatment revealed a decrease in *Lactobacillus* spp. abundance among patients diagnosed with endometritis through CD138 immunohistochemistry, while *Ralstonia* spp. and *Gardnerella* spp. were associated with CE [69]; and higher numbers of *Phyllobacterium* and *Sphingomonas* are also reported in CE patients [70]. Comparison of the intrauterine microbiome of 20 patients with polyps and CE and 10 healthy individuals identified higher proportions of bacteria such as *Bifidobacterium*, *Gardnerella, Lactobacillus*, and *Streptococcus* in the patient group and higher numbers of *Pseudomonas* in the healthy control group [71].

With increasing knowledge about bacterial communities related to CE, molecular microbiology represents a feasible tool for CE diagnosis. In fact, a new molecular diagnostic approach was described [72], and evaluation of pathogens by real-time PCR or NGS provides reliable results that are in accordance with classic diagnostic methods. Moreover, improvements in CE diagnostics and treatment may yield important changes in reproductive outcomes, as infertility conditions such as RIF are consistently related to CE [55,73,74]. Interestingly, some studies showed higher pregnancy rates and live birth rates (LBRs) in women with CE after successful treatment with antibiotics, which demonstrates the importance of microbiota management in endometritis [75,76]. Of note, besides changes in bacterial communities, variation in inflammatory signaling may also disrupt endometrial function in CE, decreasing endometrial receptivity and clinical outcomes during ART cycles [77]. Thus, further assessment of immune disruption may be needed to ensure effective treatment of uterine pathologies.

### 4.3. Repeated Implantation Failure

Shifts in endometrial microbiota are also associated with RIF occurrence. For instance, among 46 patients enrolled in ART cycles who had a history of RIF or who were attempting IVF treatment for the first time, those with RIF exhibited a significantly lower microbial diversity in endometrial fluid. Results also demonstrated higher rates of LD microbiota (>90%) and higher numbers of *Gardnerella* in RIF patients than in the control group, although not statistically significant. Additionally, *Burkholderia* was completely absent from the controls, while it was detected in 25% of RIF subjects [78]. However, one group attempted to restore LD endometrial microbiota in patients with a history of RIF, and decreased responsiveness to treatment was reported on those for whom *Gardnerella*-was the dominant bacteria [30]. Later work on the endometrial microbiota in 145 patients with RIF and 21 controls revealed similar alpha and beta diversities between groups. Nonetheless, there were different bacterial compositions, and the RIF group exhibited significant enrichment of 14 genera—including *Atopobium*, *Burkholderia*, *Delftia*, *Gardnerella*, and *Prevotella*—but similar abundance of endometrial *Lactobacillus* spp. [11]. Lastly, greater levels of *L. helveticus*, *Sneathia amnii*, and the genus *Prevotella* were reported among patients with RIF, and higher numbers of *L. iners*, *L. jensenii*, and the genus *Ralstonia* were found in subjects without RIF [79].

## 5. Tools for Endometrial Dysbiosis Management

Despite limited knowledge on the UGT microbiome, there is sufficient evidence of a shift in microbial communities in association with several gynecological and obstetrical diseases. These observations prompt testing of microbiota transplantation and antibiotic/probiotic administration to treat disorders such as BV and vulvovaginal candidiasis to restore the FGT microbiota to a potential eubiotic state [76,80,81,82].

Among methods to modify the FGT microbiota, antibiotics have the greatest number of available reports. For instance, antibiotics such as secnidazole and amoxicillin have a great impact on gynecological pathologies, as treatment led to reduced risk of bacterial vaginosis, CE resolution, and higher pregnancy and live birth rates [75,76,83,84]. Even though treatment of these conditions could improve ART outcomes, administration of antibiotics alone as a first choice to alter the microbiota remains controversial, since a wide range of action could impact not only pathogenic bacterial populations but also the proliferation of protective microbiota such as *Lactobacillus*. A meta-analysis evaluated the effects of antibiotics on clinical outcomes among patients with CE and did not observe any significant improvement in patients without confirmation of a CE cure, suggesting that CE resolution should always be confirmed before proceeding with ART. However, the same study showed that patients with cured CE had improved live birth, ongoing pregnancy, clinical pregnancy, and implantation rates compared with patients with persistent CE. Additionally, IVF results were comparable between women with cured CE and no-CE controls [74].

Administration of probiotics to induce recolonization of the genital tract has been discussed as a complementary treatment approach. For instance, probiotic supplementation with *Lactobacillus* led to better clinical outcomes than treatment with metronidazole alone in patients with BV [85], and the administration of oral and vaginal probiotics successfully improved the *Lactobacillus* content in patients with RIF [30]. Of note, the low pH of the gastrointestinal system and later need of transfer of probiotics to the appropriate colonization site through the hematogenous route or vaginal ascension are the main challenges of uterine recolonization through oral administration of probiotics [86]. Accordingly, treatment with vaginal probiotic suppository in combination with antibiotics led to more significant results if compared to the oral administration route, and this approach could significantly improve clinical outcomes in patients with repeated implantation failure [30]. Nonetheless, recent systematic reviews reported controversial results and revealed that there is no strong evidence that probiotics could improve results in women undergoing fertility treatment [87,88], although increased abundance of *Lactobacillus* species can be achieved [86].

The use of antibiotics, however, may not always be effective. For example, a patient with repeated reproductive failure after several embryo transfer cycles underwent endometrial microbiota assessment, and results revealed very low levels of *Lactobacillus* and a high abundance of *Gardnerella*. To restore *Lactobacillus* abundance, a combination treatment of antibiotics and probiotic vaginal tampons was conducted, but results demonstrated a persistent *Gardnerella* infection, and pregnancy was not achieved [89]. Hence, other strategies such as microbiota transplantation may represent promising management approaches, and its role in the treatment of BV and other gynecological conditions is reviewed elsewhere [90]. Although little is known about FGT microbiota transplantation, promising results with fecal microbiota transplantation acquired recently [91] suggest that it could be an effective treatment for gynecological and obstetrical pathologies in patients who are unresponsive to standard antibiotic/probiotic protocols. Thus, further studies should be conducted with endometrial microbiota transplantation to determine key parameters—such as dosage, method of application, and best microbiota composition—to properly select healthy donors. Although no reports have been published on endometrial microbiota transplantation, this technique could be considered as a treatment to restore microbial eubiosis in the future.

## 6. Endometrial Microbiota Composition and Reproductive Outcomes

Traditionally, the presence of bacteria in the uterine cavity was considered pathogenic, as positive cultures from endometrial samples were associated with failure to conceive and lower implantation rates [92,93]. However, the hypothesis of the non-sterile uterus and increasing evidence indicating an important role of the uterine microbiota in female health suggest that bacterial communities are crucial for pregnancy maintenance, and changes in these bacterial communities might reduce IVF success, possibly by triggering immune responses and metabolome fluctuations. Moreover, although most studies suggest that high abundance of *Lactobacillus* spp. represent the ideal environment for embryo implantation and pregnancy maintenance, there is no consensus on how the endometrial microbiota relates to reproductive outcomes (Table 1).

Considering LBRs, a prospective study including 91 women undergoing IVF treatment analyzed endometrial microbiota recovered from embryo transfer catheter tips. Positive cultures of H_2_O_2_-producing *Lactobacillus* were associated with increased LBRs, even compared with samples in which no bacteria were isolated. In contrast, recovery of *Streptococcus viridans* was associated with lower LBRs. Interestingly, no live births were reported in patients that tested positive for both *S. viridans* and H_2_O_2_-producing *Lactobacillus* in the transfer catheter [97].

Regarding pregnancy and ongoing pregnancy rates, one study reported endometrial microbiome assessments from 33 patients who underwent IVF treatment and transferred a single euploid embryo. NGS of samples collected from the distal part of the catheter demonstrated that *Lactobacillus* and *Flavobacterium* were the predominant bacterial communities in all subjects, regardless of their ongoing pregnancy outcomes [94]. Assessment of endometrial samples from 48 infertile women revealed no significant differences in endometrial diversity between patients that achieved pregnancy and those who did not get pregnant, although greater abundance of *Lactobacillus* spp., *Anaerobacillus* spp., *Burkholderia* spp., and *Gardnerella* spp. was detected in women that achieved a clinical pregnancy. In contrast, other bacteria—such as *Delftia* spp., *Prevotella* spp., *Ralstonia* spp., and *Streptococcus* spp.—were observed in higher quantities among non-pregnant women [79]. Analysis of endometrial fluid samples from 92 women undergoing IVF treatment demonstrated statistically equivalent pregnancy rates in women with an LD microbiota (≧80% *Lactobacillus*) after antibiotic/probiotic management compared with women with an NDL microbiota (<80% *Lactobacillus*), although the chances were slightly higher in the former group [96]. In a study assessing multiple reproductive outcomes with endometrial fluid samples from 35 infertile patients with RIF, NLD endometrial microbiota strongly impacted clinical outcomes during ART cycles: dysbiotic microbiota was associated with decreased implantation, pregnancy, ongoing pregnancy, and live birth rates. Additionally, all enrolled patients received a personalized embryo transfer, suggesting that results were not impacted by endometrial receptivity [13]. A similar analysis produced contrasting results. For 99 patients of Asian descent, EF samples were split into groups having eubiotic (≥80% *Lactobacillus* and *Bifidobacterium* spp.) and dysbiotic (<80% *Lactobacillus* and *Bifidobacterium* spp. with ≥20% of other bacteria) endometrial microbiota. Implantation, miscarriage, and pregnancy rates were similar between groups, suggesting that a dysbiotic uterine environment does not impact fertility [95]. Of note, the median percentage of endometrial *Lactobacillus* was significantly decreased in patients undergoing IVF treatment compared with healthy volunteers (63.90 ± 41.43% versus 99.50 ± 15.85%), and higher abundances of *Gardnerella* and *Bifidobacterium* were present in NLD samples. However, seven pregnancies were achieved in NLD cases without any microbiome management intervention, and five of these were ongoing pregnancies by the time of the report [16]. Similarly, other studies reported cases of normal pregnancies with very low *Lactobacillus* abundance, including patients in which these bacteria were completely absent [22,26,95].

Interestingly, a significant change in endometrial microbiota was reported in early pregnancy compared with samples collected before a clinical miscarriage in the same woman. Endometrial fluid samples collected previous to spontaneous miscarriage revealed an NLD profile, in which *Lactobacilli* accounted for 15% of the uterine bacterial composition, and a significant diversity of bacterial genera—such as *Streptococcus*, *Pseudomonas*, and *Propionibacterium*—was present. However, a sample collected at the fourth week of a successful gestation in the same patient showed a significant decrease in bacterial diversity, with *Lactobacillus* dominance accounting for 91% of the sample’s microbiota [98].

Corroborating these findings, a prospective observational multicenter study including 342 infertile patients demonstrated that higher abundance of *Lactobacillus* strongly correlated to LBR, suggesting that *Lactobacillus* could be a biomarker for treatment success [18]. However, the presence of *Atopobium*, *Bifidobacterium*, *Chryseobacterium*, *Gardnerella*, *Haemophilus*, *Klebsiella*, *Neisseria*, *Staphylococcus*, and *Streptococcus* was associated with worse prognosis after ART treatment (e.g., no pregnancy, biochemical pregnancy, or clinical miscarriage). For instance, *Gardnerella*, *Klebsiella*, and *Streptococcus* were significantly increased in non-pregnant patients, *Klebsisella* and *Staphylococcus* were related to clinical miscarriage, and *Enterococcus* was enriched in subjects that had a biochemical pregnancy [18].

While most studies have focused on describing the effects of individualized bacterial genera or species, some studies have reported tight regulation in the FGT microbiota that suggests complex interplay between bacterial communities [18,19]. Thus, it is feasible to speculate that clinical outcomes in ART treatment are instead affected by the combination of all bacterial communities present in the uterine cavity and how they interact with host tissue, which ultimately suggests that similar bacterial communities may lead to different outcomes in distinct patients. Accordingly, a healthy endometrial microbiota could be considered a composition of distinct communities that are permissive for embryo implantation and pregnancy maintenance despite a minor presence of pathogenic bacteria.

## 7. Challenges of Studying the Endometrial Microbiota

The reported lack of consensus on the endometrial microbiota taxonomic core could be due to several limitations in microbiome studies. Unstandardized methodological practices and important differences in study populations make available studies difficult to compare, and invasive sampling methods are major limitations to increasing sample cohort sizes. Taking into consideration the current lack of large cohort studies on the endometrial microbiome, comparability between studies is needed, and the establishment of general protocols would allow this kind of analysis.

### 7.1. Population and Study Design

The sample cohort is one of the biggest limitations on endometrial microbiome studies. Patient characteristics—such as ethnicity, age, and sexual habits—should be carefully considered during the process of cohort selection, as several studies have suggested that these characteristics may be responsible for community fluctuations in the FGT, including the uterus [19,25,28]. For instance, important taxa dissimilarities were observed between patients of different genetic backgrounds, suggesting that black women harbor a distinct FGT microbiome [19,99,100]. These differences among populations may represent a significant bias in population selection and should, therefore, be carefully conducted.

Strict inclusion and exclusion criteria considering hormonal treatment, antibiotic/probiotic administration, and intrauterine devices should also be used because these interventions may influence results through changes in endometrial microbiome composition [17,27,29,30]. Thus, study designs should consider different demographic and health parameters to mitigate inter- and intraindividual dissimilarity and allow results to be extrapolated to wider populations of infertile patients.

Aside from interindividual dissimilarity, intrapersonal variability may also account for differences among studies. Because there are reports of microbiota changes within the menstrual cycle [9,19,30], collection of samples on specific days after menses could also make different studies more easily comparable. For instance, studies assessing the impact of UGT microbiota in ART treatment should acquire samples under conditions equivalent to those found at the period of embryo implantation, avoiding bias mainly related to hormonal swings.

Acquiring uterine samples, sometimes through surgical procedures such as hysterectomy or laparoscopy, is highly invasive and is performed mainly when there are underlying uterine pathologies—such as cancer, endometriosis, and fibroids. Consequently, most available studies include small cohorts of individuals, usually around 30–60 patients, which directly impacts the statistical power of the analysis [101]. Furthermore, enrollment of healthy controls remains a challenge. Although some patients undergoing IVF treatment may not bear these conditions, they cannot be considered as healthy controls due to their infertility status. Thus, inclusion criteria restricted to male factor infertility or fertile women undergoing tubal sterilization could lead to a more controlled study population and a selected control group.

### 7.2. Sample Handling and Experimental Design

In most studies, endometrial samples are usually collected through the vaginal–cervical route during gynecological assessment or embryo transfers. Thus, there is a risk of contamination with vaginal microbiota during sampling that could impact microbiome analyses, especially considering the low-biomass microbiome found in the uterus and the different bacterial communities found throughout the FGT [9,16,28,79]. For instance, in one study only 2 of 26 pairs of samples of endometrial fluid and vaginal aspirates had the same bacterial operational taxonomic units (OTUs), while six paired samples had completely different microbial communities [13]. These results suggest that the uterus has its own microbial communities, and any kind of contamination from the lower genital tract could result in significant misrepresentation of the endometrial microbiota. Although invasive procedures such as laparoscopy and hysterectomy avoid vaginal contamination, they can only be performed on individuals that bear underlying conditions, which directly impairs patient recruitment. Of note, different sampling tools, such as double-sheathed embryo transfer catheters and curettes connected to a syringe, were developed to decrease contamination and allowed for the collection of a significant amount of material [72,94,102].

Importantly, different microbiome compositions within uterine samples has been observed, and studies suggest that endometrial fluid and endometrial biopsy (EB) samples might not contain the same bacterial diversity. For instance, among the 10 most abundant species in both types of samples, taxa such as Enterobacteriaceae, *Staphylococcus*, and *Stenotrophomonas* were differentially present. Furthermore, several taxa could only be found in either endometrial biopsy or endometrial fluid, including *Achromobacter, Brevundimonas,* and Verrucomicrobiaceae [103]. Similar results were reported by other groups, as *Streptomyces* and *Clostridium* were detected only in EF, while *Klebsiella* and *Micrococcus* were exclusively detected in EB samples. Still, despite different microbiota compositions, dysbiotic states both in EB and EF led to equivalent clinical outcomes [18]. These variations in endometrial samples ultimately represent the need for another layer of standardization on experimental design to facilitate comparisons among uterine microbiome studies.

Furthermore, the presence of bacterial DNA in laboratory reagents, plastic consumables, and protective equipment could also significantly impact analyses of bacteria communities in the endometrium [104]. Although it is difficult to control all sources of contamination, negative and blank controls should be carefully processed to detect basal contaminants, and the use of three different negative controls and two positive controls may be important to optimize the detection of contaminants [101]. 

Technical diversity in experimental design can also be considered a source of inconsistency among studies. After collection, samples should be properly stored at low temperatures or specific stabilizing solutions to prevent DNA degradation, and inefficiency in this step can lead to shifts in the composition of the microbiota. Different DNA extraction kits, pair of primers used, and sequencing platforms may also render greater differences between studies, and standardized approaches should be considered to facilitate study comparison. For instance, analysis of the V1 region better differentiates *Staphylococcus* species, while V2 and V3 would be more suitable for studying *Mycobacterium* and *Haemophilus* species, respectively [105]. Importantly, species that impact female health, such as *Gardnerella vaginalis* and *Chlamydia trachomatis*, may not be properly assessed in sequencing based on V1/V2 regions [106]. Altogether, these data demonstrate the significant variability in results caused by the choice of different hypervariable regions. As under- or overestimation of taxa impacts analyses of endometrial microbiota diversity, careful considerations should be taken on this matter.

## 8. Concluding Remarks

Despite growing evidence of the impact of microbiota on gynecological and obstetrical conditions, there is still no consensus on the endometrial bacterial core. Furthermore, standardized protocols and larger patient cohorts are required for studies to be comparable and to help understand the physiological uterine microbiota, as well as how dysbiosis could impact clinical outcomes. Importantly, responses from the host also modulate many aspects of human conception, and future studies unraveling the mechanisms of microbiota–host interactions may highlight important topics on how bacterial communities drive infertility. We propose that a physiological and healthy endometrial microbiota should be considered a group of microorganisms that is permissive for embryo implantation and live birth, regardless of the minimum presence of pathogenic bacteria.

## Figures and Tables

**Table 1:** Studies associating the uterine microbiome with reproductive outcomes ^a^.

Authors	Population	Sample Size	Average Age (Years)	Ethnicity	Sampling	Microbiome Analysis	Microbiota Findings	Reference
Diaz-Martinez et al., 2021	Women undergoing IVF with frozen-thawed euploid ET	Total: 48 CP: 21 NP: 27	39.4	Not reported, conducted in Spain	Tao Brush IUMC Endometrial Sampler	Illumina^®^ MiSeq ™ system platform for NGS of 16S rRNA gene—hypervariable region V3-V4	Pregnant and non-pregnant women with comparable alpha and beta diversity of endometrial microbiota.	[79]
Franasiak et al., 2016	Women undergoing single euploid ET	Total: 33 OGP: 18 NP: 15	35.9	79% Caucasian 15% Asian 3% African American 3% Hispanic	Distal portion of ET catheter tip	Ion Torrent NGS of 16S rRNA gene —hypervariable regions V2-4-8 and V3-6, 7-9	No differences between OGP and no OGP.	[94]
Hashimoto et al., 2019	Women undergoing thawed ET	Total: 99 CP: 53 OGP: 40 CM: 5 NP: 46	35.2	Japanese	EF samples aspirated with a Kitazato IUI catheter	Illumina^®^ MiSeq ™ system platform for NGS of 16S rRNA gene —hypervariable region V4	Pregnancy, implantation, and miscarriage rates were comparable between LD and NLD groups. Pregnancy in patients with complete absence of *Lactobacillus* was reported.	[95]
Kyono et al., 2018	Women undergoing thawed ET	Total: 92 CP: 50 CM: 11 NP: 42	36.9	Asian: 90 Japanese 1 Korean 1 Chinese	EF samples collected using Kitazato IUI catheter	Illumina^®^ MiSeq ™ system platform for NGS of 16S rRNA gene —hypervariable region V4	Pregnancy rates slightly higher in LDM group, although the difference was not statistically significant.	[96]
Moore et al., 2000	Women undergoing IVF treatment	Total: 91 LB: 27 No LB: 64	35.3	Not reported, conducted in the USA	Distal portion of ET catheter tip	Culture plates and biochemical tests	Increased LB rates associated with recovery of H_2_O_2_-producing *Lactobacillus*. Decreased LB rate when samples were positive for *Streptococcus viridans*.	[97]
Moreno et al., 2016	Infertile women undergoing ART treatment	Total: 32 LB: 12 CM: 5 NP: 15	39.3	Not reported, conducted in Spain	EF was aspirated using Wallace catheter	454 pyrosequencing of 16S rRNA gene—hypervariable region V3-V5	NLD endometrial microbiota associated with poorer clinical outcomes (decreased implantation, pregnancy, OGP, and LB rates).	[13]
Moreno et al., 2020	Patient with primary infertility and previous unsuccessful IVF cycles	One woman (case report)	28	Not reported, conducted in Spain	EF was aspirated with a double lumen embryo transfer catheter	Ion S5 XL system for NGS of 16S rRNA gene—hypervariable regions V2-4-8 and V3-6, 7-9	Higher bacterial community diversity and lower *Lactobacillus* abundance before spontaneous abortion. Increased numbers of *Lactobacillus* before the healthy pregnancy, as *Lactobacillus iners* was the most prevalent bacteria.	[98]
Moreno et al., 2021	Infertile women undergoing ART treatment	Total: 342 LB: 141 BP: 27 CM: 28 EP: 2 NP: 144	36.0	57.3% Caucasian 14.0% East Asian 11.4% Hispanic 17.3% others	EF aspirated through catheter attached to a syringe. EB performed with Pipelle catheter	Ion S5 XL system for NGS of 16S rRNA gene—hypervariable regions V2-4-8 and V3-6, 7-9	Increased abundance of *Lactobacillus* consistently associated with higher LB rates. Dysbiotic microbial state in the uterus composed of *Atopobium*, *Bifidobacteirum*, *Gardnerella*, *Klesbiella*, *Streptococcus*, and others was associated with poorer clinical outcomes.	[18]

^a^ ART, assisted reproductive technologies; CM, clinical miscarriage; CP, clinical pregnancy; EB, endometrial biopsy; EF, endometrial fluid; EP, ectopic pregnancy; ET, embryo transfer; IUI, intrauterine insemination; IVF, in vitro fertilization; LB, live birth; LD, *Lactobacillus* dominated; NGS, next-generation sequencing; NLD, non-*Lactobacillus* dominated; NP, no pregnancy; OGP, ongoing pregnancy.

## References

[1] Lederberg J., McCray A.T. ‘Ome Sweet’ Omics—A Genealogical Treasury of Words. https://lhncbc.nlm.nih.gov/LHC-publications/pubs/OmeSweetOmicsAGenealogicalTreasuryofWords.html.

[2] Peterson J., Garges S., Giovanni M., McInnes P., Wang L., Schloss J.A., Bonazzi V., McEwen J.E., Wetterstrand K.A., Deal C. (2009). The NIH Human Microbiome Project. Genome Res..

[3] Baker J.M., Chase D.M., Herbst-Kralovetz M.M. (2018). Uterine Microbiota: Residents, Tourists, or Invaders?. Front. Immunol..

[4] Perez-Muñoz M.E., Arrieta M.-C., Ramer-Tait A.E., Walter J. (2017). A Critical Assessment of the “Sterile Womb” and “in Utero Colonization” Hypotheses: Implications for Research on the Pioneer Infant Microbiome. Microbiome.

[5] Benner M., Ferwerda G., Joosten I., van der Molen R.G. (2018). How Uterine Microbiota Might Be Responsible for a Receptive, Fertile Endometrium. Hum. Reprod. Update.

[6] Sparks R.A., Purrier B.G., Watt P.J., Elstein M. (1981). Bacteriological Colonisation of Uterine Cavity: Role of Tailed Intrauterine Contraceptive Device. BMJ.

[7] Pereira N., Hutchinson A.P., Lekovich J.P., Hobeika E., Elias R.T. (2016). Antibiotic Prophylaxis for Gynecologic Procedures Prior to and during the Utilization of Assisted Reproductive Technologies: A Systematic Review. J. Pathog..

[8] Svenstrup H.F., Fedder J., Abraham-Peskir J., Birkelund S., Christiansen G. (2003). Mycoplasma Genitalium Attaches to Human Spermatozoa. Hum. Reprod..

[9] Chen C., Song X., Wei W., Zhong H., Dai J., Lan Z., Li F., Yu X., Feng Q., Wang Z. (2017). The Microbiota Continuum along the Female Reproductive Tract and Its Relation to Uterine-Related Diseases. Nat. Commun..

[10] Wee B.A., Thomas M., Sweeney E.L., Frentiu F.D., Samios M., Ravel J., Gajer P., Myers G., Timms P., Allan J.A. (2018). A Retrospective Pilot Study to Determine Whether the Reproductive Tract Microbiota Differs between Women with a History of Infertility and Fertile Women. Aust. N. Z. J. Obstet. Gynaecol..

[11] Ichiyama T., Kuroda K., Nagai Y., Urushiyama D., Ohno M., Yamaguchi T., Nagayoshi M., Sakuraba Y., Yamasaki F., Hata K. (2021). Analysis of Vaginal and Endometrial Microbiota Communities in Infertile Women with a History of Repeated Implantation Failure. Reprod. Med. Biol..

[12] Mitchell C.M., Haick A., Nkwopara E., Garcia R., Rendi M., Agnew K., Fredricks D.N., Eschenbach D. (2015). Colonization of the Upper Genital Tract by Vaginal Bacterial Species in Nonpregnant Women. Am. J. Obstet. Gynecol..

[13] Moreno I., Codoñer F.M., Vilella F., Valbuena D., Martinez-Blanch J.F., Jimenez-Almazán J., Alonso R., Alamá P., Remohí J., Pellicer A. (2016). Evidence That the Endometrial Microbiota Has an Effect on Implantation Success or Failure. Am. J. Obstet. Gynecol..

[14] Verstraelen H., Vilchez-Vargas R., Desimpel F., Jauregui R., Vankeirsbilck N., Weyers S., Verhelst R., de Sutter P., Pieper D.H., van de Wiele T. (2016). Characterization of the Human Uterine Microbiome in Non-Pregnant Women through Deep Sequencing of the V1-2 Region of the 16S RRNA Gene. PeerJ.

[15] Tao X., Franasiak J.M., Zhan Y., Scott R.T., Rajchel J., Bedard J., Newby R., Scott R.T., Treff N.R., Chu T. (2017). Characterizing the Endometrial Microbiome by Analyzing the Ultra-Low Bacteria from Embryo Transfer Catheter Tips in IVF Cycles: Next Generation Sequencing (NGS) Analysis of the 16S Ribosomal Gene. Hum. Microbiome J..

[16] Kyono K., Hashimoto T., Nagai Y., Sakuraba Y. (2018). Analysis of Endometrial Microbiota by 16S Ribosomal RNA Gene Sequencing among Infertile Patients: A Single-Center Pilot Study. Reprod. Med. Biol..

[17] Carosso A., Revelli A., Gennarelli G., Canosa S., Cosma S., Borella F., Tancredi A., Paschero C., Boatti L., Zanotto E. (2020). Controlled Ovarian Stimulation and Progesterone Supplementation Affect Vaginal and Endometrial Microbiota in IVF Cycles: A Pilot Study. J. Assist. Reprod. Genet..

[18] Moreno I., Garcia-Grau I., Perez-Villaroya D., Gonzalez- M., Bahçeci M., Barrionuevo M.J., Taguchi S., Puente E., Dimattina M., Wei Lim M. (2021). Endometrial Microbiota Composition Is Associated with Reproductive Outcome in Infertile Patients. medRxiv.

[19] Gajer P., Brotman R.M., Bai G., Sakamoto J., Schütte U.M.E., Zhong X., Koenig S.S.K., Fu L., Ma Z., Zhou X. (2012). Temporal Dynamics of the Human Vaginal Microbiota. Sci. Transl. Med..

[20] Li F., Chen C., Wei W., Wang Z., Dai J., Hao L., Song L., Zhang X., Zeng L., Du H. (2018). The Metagenome of the Female Upper Reproductive Tract. GigaScience.

[21] Winters A.D., Romero R., Gervasi M.T., Gomez-Lopez N., Tran M.R., Garcia-Flores V., Pacora P., Jung E., Hassan S.S., Hsu C.-D. (2019). Does the Endometrial Cavity Have a Molecular Microbial Signature?. Sci. Rep..

[22] Leoni C., Ceci O., Manzari C., Fosso B., Volpicella M., Ferrari A., Fiorella P., Pesole G., Cicinelli E., Ceci L.R. (2019). Human Endometrial Microbiota at Term of Normal Pregnancies. Genes.

[23] Sola-Leyva A., Andrés-León E., Molina N.M., Terron-Camero L.C., Plaza-Díaz J., Sáez-Lara M.J., Gonzalvo M.C., Sánchez R., Ruíz S., Martínez L. (2021). Mapping the Entire Functionally Active Endometrial Microbiota. Hum. Reprod..

[24] Miles S.M., Hardy B.L., Merrell D.S. (2017). Investigation of the Microbiota of the Reproductive Tract in Women Undergoing a Total Hysterectomy and Bilateral Salpingo-Oopherectomy. Fertil. Steril..

[25] Wang J., Li Z., Ma X., Du L., Jia Z., Cui X., Yu L., Yang J., Xiao L., Zhang B. (2021). Translocation of Vaginal Microbiota Is Involved in Impairment and Protection of Uterine Health. Nat. Commun..

[26] Riganelli L., Iebba V., Piccioni M., Illuminati I., Bonfiglio G., Neroni B., Calvo L., Gagliardi A., Levrero M., Merlino L. (2020). Structural Variations of Vaginal and Endometrial Microbiota: Hints on Female Infertility. Front. Cell. Infect. Microbiol..

[27] Achilles S.L., Austin M.N., Meyn L.A., Mhlanga F., Chirenje Z.M., Hillier S.L. (2018). Impact of Contraceptive Initiation on Vaginal Microbiota. Am. J. Obstet. Gynecol..

[28] Pelzer E.S., Willner D., Buttini M., Huygens F. (2018). A Role for the Endometrial Microbiome in Dysfunctional Menstrual Bleeding. Antonie Leeuwenhoek.

[29] Brooks J.P., Edwards D.J., Blithe D.L., Fettweis J.M., Serrano M.G., Sheth N.U., Strauss J.F., Buck G.A., Jefferson K.K. (2017). Effects of Combined Oral Contraceptives, Depot Medroxyprogesterone Acetate and the Levonorgestrel-Releasing Intrauterine System on the Vaginal Microbiome. Contraception.

[30] Kadogami D., Nakaoka Y., Morimoto Y. (2020). Use of a Vaginal Probiotic Suppository and Antibiotics to Influence the Composition of the Endometrial Microbiota. Reprod. Biol..

[31] Kovachev S. (2018). Defence Factors of Vaginal Lactobacilli. Crit. Rev. Microbiol..

[32] Amabebe E., Anumba D.O.C. (2018). The Vaginal Microenvironment: The Physiologic Role of Lactobacilli. Front. Med..

[33] Yeoman C.J., Yildirim S., Thomas S.M., Durkin A.S., Torralba M., Sutton G., Buhay C.J., Ding Y., Dugan-Rocha S.P., Muzny D.M. (2010). Comparative Genomics of Gardnerella Vaginalis Strains Reveals Substantial Differences in Metabolic and Virulence Potential. PLoS ONE.

[34] Sirota I., Zarek S., Segars J. (2014). Potential Influence of the Microbiome on Infertility and Assisted Reproductive Technology. Semin. Reprod. Med..

[35] Muzny C.A., Blanchard E., Taylor C.M., Aaron K.J., Talluri R., Griswold M.E., Redden D.T., Luo M., Welsh D.A., van der Pol W.J. (2018). Identification of Key Bacteria Involved in the Induction of Incident Bacterial Vaginosis: A Prospective Study. J. Infect. Dis..

[36] Jung H.-S., Ehlers M.M., Lombaard H., Redelinghuys M.J., Kock M.M. (2017). Etiology of Bacterial Vaginosis and Polymicrobial Biofilm Formation. Crit. Rev. Microbiol..

[37] Miller E.A., Beasley D.E., Dunn R.R., Archie E.A. (2016). Lactobacilli Dominance and Vaginal PH: Why Is the Human Vaginal Microbiome Unique?. Front. Microbiol..

[38] Azkargorta M., Escobes I., Iloro I., Osinalde N., Corral B., Ibañez-Perez J., Exposito A., Prieto B., Elortza F., Matorras R. (2018). Differential Proteomic Analysis of Endometrial Fluid Suggests Increased Inflammation and Impaired Glucose Metabolism in Non-Implantative IVF Cycles and Pinpoints PYGB as a Putative Implantation Marker. Hum. Reprod..

[39] Azkargorta M., Bregón-Villahoz M., Escobes I., Ibáñez-Pérez J., Iloro I., Iglesias M., Diez-Zapirain M., Rabanal A., Prieto B., Moragues M.-D. (2020). In-Depth Proteomics and Natural Peptidomics Analyses Reveal Antibacterial Peptides in Human Endometrial Fluid. J. Proteom..

[40] Frew L., Stock S.J. (2011). Antimicrobial Peptides and Pregnancy. Reproduction.

[41] Reddy K.V.R., Yedery R.D., Aranha C. (2004). Antimicrobial Peptides: Premises and Promises. Int. J. Antimicrob. Agents.

[42] Fu M., Zhang X., Liang Y., Lin S., Qian W., Fan S. (2020). Alterations in Vaginal Microbiota and Associated Metabolome in Women with Recurrent Implantation Failure. mBio.

[43] Pruski P., Correia G.D.S., Lewis H.v., Capuccini K., Inglese P., Chan D., Brown R.G., Kindinger L., Lee Y.S., Smith A. (2021). Direct On-Swab Metabolic Profiling of Vaginal Microbiome Host Interactions during Pregnancy and Preterm Birth. Nat. Commun..

[44] Kitaya K., Tada Y., Hayashi T., Taguchi S., Funabiki M., Nakamura Y. (2014). Comprehensive Endometrial Immunoglobulin Subclass Analysis in Infertile Women Suffering from Repeated Implantation Failure with or without Chronic Endometritis. Am. J. Reprod. Immunol..

[45] Tseng J.F., Ryan I.P., Milam T.D., Murai J.T., Schriock E.D., Landers D.v, Taylor R.N. (1996). Interleukin-6 Secretion in Vitro Is up-Regulated in Ectopic and Eutopic Endometrial Stromal Cells from Women with Endometriosis. J. Clin. Endocrinol. Metab..

[46] Sikora J., Mielczarek-Palacz A., Kondera-Anasz Z. (2016). Association of the Precursor of Interleukin-1β and Peritoneal Inflammation-Role in Pathogenesis of Endometriosis. J. Clin. Lab. Anal..

[47] Jess T., Frisch M., Jørgensen K.T., Pedersen B.V., Nielsen N.M. (2012). Increased Risk of Inflammatory Bowel Disease in Women with Endometriosis: A Nationwide Danish Cohort Study. Gut.

[48] Robertson S.A., Care A.S., Skinner R.J. (2007). Interleukin 10 Regulates Inflammatory Cytokine Synthesis to Protect Against Lipopolysaccharide-Induced Abortion and Fetal Growth Restriction in Mice. Biol. Reprod..

[49] Guerin L.R., Prins J.R., Robertson S.A. (2009). Regulatory T-Cells and Immune Tolerance in Pregnancy: A New Target for Infertility Treatment?. Hum. Reprod. Update.

[50] Shima T., Sasaki Y., Itoh M., Nakashima A., Ishii N., Sugamura K., Saito S. (2010). Regulatory T Cells Are Necessary for Implantation and Maintenance of Early Pregnancy but Not Late Pregnancy in Allogeneic Mice. J. Reprod. Immunol..

[51] Barnea E.R., Vialard F., Moindjie H., Ornaghi S., Dieudonne M.N., Paidas M.J. (2016). PreImplantation Factor (PIF*) Endogenously Prevents Preeclampsia: Promotes Trophoblast Invasion and Reduces Oxidative Stress. J. Reprod. Immunol..

[52] Dimitriadis E., White C.A., Jones R.L., Salamonsen L.A. (2005). Cytokines, Chemokines and Growth Factors in Endometrium Related to Implantation. Hum. Reprod. Update.

[53] Satoh T., Akira S. (2016). Toll-Like Receptor Signaling and Its Inducible Proteins. Microbiol. Spectr..

[54] Koga K., Izumi G., Mor G., Fujii T., Osuga Y. (2014). Toll-like Receptors at the Maternal-Fetal Interface in Normal Pregnancy and Pregnancy Complications. Am. J. Reprod. Immunol..

[55] Johnston-MacAnanny E.B., Hartnett J., Engmann L.L., Nulsen J.C., Sanders M.M., Benadiva C.A. (2010). Chronic Endometritis Is a Frequent Finding in Women with Recurrent Implantation Failure after in Vitro Fertilization. Fertil. Steril..

[56] Punzón-Jiménez P., Labarta E. (2021). The Impact of the Female Genital Tract Microbiome in Women Health and Reproduction: A Review. J. Assist. Reprod. Genet..

[57] Haggerty C.L., Totten P.A., Tang G., Astete S.G., Ferris M.J., Norori J., Bass D.C., Martin D.H., Taylor B.D., Ness R.B. (2016). Identification of Novel Microbes Associated with Pelvic Inflammatory Disease and Infertility. Sex. Transm. Infect..

[58] Ravel J., Moreno I., Simón C. (2021). Bacterial Vaginosis and Its Association with Infertility, Endometritis, and Pelvic Inflammatory Disease. Am. J. Obstet. Gynecol..

[59] Haggerty C.L., Hillier S.L., Bass D.C., Ness R.B. (2004). Bacterial Vaginosis and Anaerobic Bacteria Are Associated with Endometritis. Clin. Infect. Dis..

[60] Khan K.N., Fujishita A., Kitajima M., Hiraki K., Nakashima M., Masuzaki H. (2014). Intra-Uterine Microbial Colonization and Occurrence of Endometritis in Women with Endometriosis. Hum. Reprod..

[61] Khan K.N., Fujishita A., Masumoto H., Muto H., Kitajima M., Masuzaki H., Kitawaki J. (2016). Molecular Detection of Intrauterine Microbial Colonization in Women with Endometriosis. Eur. J. Obstet. Gynecol. Reprod. Biol..

[62] Wessels J.M., Domínguez M.A., Leyland N.A., Agarwal S.K., Foster W.G. (2021). Endometrial Microbiota Is More Diverse in People with Endometriosis than Symptomatic Controls. Sci. Rep..

[63] Hernandes C., Silveira P., Rodrigues Sereia A.F., Christoff A.P., Mendes H., Valter de Oliveira L.F., Podgaec S. (2020). Microbiome Profile of Deep Endometriosis Patients: Comparison of Vaginal Fluid, Endometrium and Lesion. Diagnostics.

[64] Cregger M.A., Lenz K., Leary E., Leach R., Fazleabas A., White B., Braundmeier A. (2017). Reproductive Microbiomes: Using the Microbiome as a Novel Diagnostic Tool for Endometriosis. Reprod. Immunol. Open Access.

[65] Wei W., Zhang X., Tang H., Zeng L., Wu R. (2020). Microbiota Composition and Distribution along the Female Reproductive Tract of Women with Endometriosis. Ann. Clin. Microbiol. Antimicrob..

[66] Leonardi M., Hicks C., El-Assaad F., El-Omar E., Condous G. (2020). Endometriosis and the Microbiome: A Systematic Review. BJOG Int. J. Obstet. Gynaecol..

[67] Cicinelli E., de Ziegler D., Nicoletti R., Colafiglio G., Saliani N., Resta L., Rizzi D., de Vito D. (2008). Chronic Endometritis: Correlation among Hysteroscopic, Histologic, and Bacteriologic Findings in a Prospective Trial with 2190 Consecutive Office Hysteroscopies. Fertil. Steril..

[68] Liu Y., Ko E.Y.-L., Wong K.K.-W., Chen X., Cheung W.-C., Law T.S.-M., Chung J.P.-W., Tsui S.K.-W., Li T.-C., Chim S.S.-C. (2019). Endometrial Microbiota in Infertile Women with and without Chronic Endometritis as Diagnosed Using a Quantitative and Reference Range-Based Method. Fertil. Steril..

[69] Lozano F.M., Bernabeu A., Lledo B., Morales R., Diaz M., Aranda F.I., Llacer J., Bernabeu R. (2021). Characterization of the Vaginal and Endometrial Microbiome in Patients with Chronic Endometritis. Eur. J. Obstet. Gynecol. Reprod. Biol..

[70] Chen P., Chen P., Guo Y., Fang C., Li T. (2021). Interaction Between Chronic Endometritis Caused Endometrial Microbiota Disorder and Endometrial Immune Environment Change in Recurrent Implantation Failure. Front. Immunol..

[71] Fang R.-L., Chen L.-X., Shu W.-S., Yao S.-Z., Wang S.-W., Chen Y.-Q. (2016). Barcoded Sequencing Reveals Diverse Intrauterine Microbiomes in Patients Suffering with Endometrial Polyps. Am. J. Transl. Res..

[72] Moreno I., Cicinelli E., Garcia-Grau I., Gonzalez-Monfort M., Bau D., Vilella F., de Ziegler D., Resta L., Valbuena D., Simon C. (2018). The Diagnosis of Chronic Endometritis in Infertile Asymptomatic Women: A Comparative Study of Histology, Microbial Cultures, Hysteroscopy, and Molecular Microbiology. Am. J. Obstet. Gynecol..

[73] Kitaya K., Takeuchi T., Mizuta S., Matsubayashi H., Ishikawa T. (2018). Endometritis: New Time, New Concepts. Fertil. Steril..

[74] Vitagliano A., Saccardi C., Noventa M., di Spiezio Sardo A., Saccone G., Cicinelli E., Pizzi S., Andrisani A., Litta P.S. (2018). Effects of Chronic Endometritis Therapy on in Vitro Fertilization Outcome in Women with Repeated Implantation Failure: A Systematic Review and Meta-Analysis. Fertil. Steril..

[75] Cicinelli E., Matteo M., Tinelli R., Lepera A., Alfonso R., Indraccolo U., Marrocchella S., Greco P., Resta L. (2015). Prevalence of Chronic Endometritis in Repeated Unexplained Implantation Failure and the IVF Success Rate after Antibiotic Therapy. Hum. Reprod..

[76] Cicinelli E., Matteo M., Trojano G., Mitola P.C., Tinelli R., Vitagliano A., Crupano F.M., Lepera A., Miragliotta G., Resta L. (2018). Chronic Endometritis in Patients with Unexplained Infertility: Prevalence and Effects of Antibiotic Treatment on Spontaneous Conception. Am. J. Reprod. Immunol..

[77] Park H.J., Kim Y.S., Yoon T.K., Lee W.S. (2016). Chronic Endometritis and Infertility. Clin. Exp. Reprod. Med..

[78] Kitaya K., Nagai Y., Arai W., Sakuraba Y., Ishikawa T. (2019). Characterization of Microbiota in Endometrial Fluid and Vaginal Secretions in Infertile Women with Repeated Implantation Failure. Mediat. Inflamm..

[79] Del Diaz-Martínez M.C., Bernabeu A., Lledó B., Carratalá-Munuera C., Quesada J.A., Lozano F.M., Ruiz V., Morales R., Llácer J., Ten J. (2021). Impact of the Vaginal and Endometrial Microbiome Pattern on Assisted Reproduction Outcomes. J. Clin. Med..

[80] Falagas M.E. (2006). Probiotics for Prevention of Recurrent Vulvovaginal Candidiasis: A Review. J. Antimicrob. Chemother..

[81] Wang Z., He Y., Zheng Y. (2019). Probiotics for the Treatment of Bacterial Vaginosis: A Meta-Analysis. Int. J. Environ. Res. Public Health.

[82] Lev-Sagie A., Goldman-Wohl D., Cohen Y., Dori-Bachash M., Leshem A., Mor U., Strahilevitz J., Moses A.E., Shapiro H., Yagel S. (2019). Vaginal Microbiome Transplantation in Women with Intractable Bacterial Vaginosis. Nat. Med..

[83] Abd El Aziz M.A., Sharifipour F., Abedi P., Jahanfar S., Judge H.M. (2019). Secnidazole for Treatment of Bacterial Vaginosis: A Systematic Review. BMC Womens Health.

[84] Cicinelli E., Matteo M., Tinelli R., Pinto V., Marinaccio M., Indraccolo U., de Ziegler D., Resta L. (2014). Chronic Endometritis Due to Common Bacteria Is Prevalent in Women With Recurrent Miscarriage as Confirmed by Improved Pregnancy Outcome After Antibiotic Treatment. Reprod. Sci..

[85] Recine N., Palma E., Domenici L., Giorgini M., Imperiale L., Sassu C., Musella A., Marchetti C., Muzii L., Benedetti Panici P. (2016). Restoring Vaginal Microbiota: Biological Control of Bacterial Vaginosis. A Prospective Case–Control Study Using Lactobacillus Rhamnosus BMX 54 as Adjuvant Treatment against Bacterial Vaginosis. Arch. Gynecol. Obstet..

[86] López-Moreno A., Aguilera M. (2021). Vaginal Probiotics for Reproductive Health and Related Dysbiosis: Systematic Review and Meta-Analysis. J. Clin. Med..

[87] Jarde A., Lewis-Mikhael A.-M., Moayyedi P., Stearns J.C., Collins S.M., Beyene J., McDonald S.D. (2018). Pregnancy Outcomes in Women Taking Probiotics or Prebiotics: A Systematic Review and Meta-Analysis. BMC Pregnancy Childbirth.

[88] Corbett G., Crosby D., McAuliffe F. (2021). Probiotic Therapy in Couples with Infertility: A Systematic Review. Eur. J. Obstet. Gynecol. Reprod. Biol..

[89] Garcia-Grau I., Perez-Villaroya D., Bau D., Gonzalez-Monfort M., Vilella F., Moreno I., Simon C. (2019). Taxonomical and Functional Assessment of the Endometrial Microbiota in A Context of Recurrent Reproductive Failure: A Case Report. Pathogens.

[90] Ma D., Chen Y., Chen T. (2019). Vaginal Microbiota Transplantation for the Treatment of Bacterial Vaginosis: A Conceptual Analysis. FEMS Microbiol. Lett..

[91] Wang J.-W., Kuo C.-H., Kuo F.-C., Wang Y.-K., Hsu W.-H., Yu F.-J., Hu H.-M., Hsu P.-I., Wang J.-Y., Wu D.-C. (2019). Fecal Microbiota Transplantation: Review and Update. J. Formos. Med. Assoc..

[92] Salim R., Ben-Shlomo I., Colodner R., Keness Y., Shalev E. (2002). Bacterial Colonization of the Uterine Cervix and Success Rate in Assisted Reproduction: Results of a Prospective Survey. Hum. Reprod..

[93] Egbase P.E., Al-Sharhan M., Al-Othman S., Al-Mutawa M., Udo E.E., Grudzinskas J.G. (1996). Fertilization and Early Embryology: Incidence of Microbial Growth from the Tip of the Embryo Transfer Catheter after Embryo Transfer in Relation to Clinical Pregnancy Rate Following in-Vitro Fertilization and Embryo Transfer. Hum. Reprod..

[94] Franasiak J.M., Werner M.D., Juneau C.R., Tao X., Landis J., Zhan Y., Treff N.R., Scott R.T. (2016). Endometrial Microbiome at the Time of Embryo Transfer: Next-Generation Sequencing of the 16S Ribosomal Subunit. J. Assist. Reprod. Genet..

[95] Hashimoto T., Kyono K. (2019). Does Dysbiotic Endometrium Affect Blastocyst Implantation in IVF Patients?. J. Assist. Reprod. Genet..

[96] Kyono K., Hashimoto T., Kikuchi S., Nagai Y., Sakuraba Y. (2018). A Pilot Study and Case Reports on Endometrial Microbiota and Pregnancy Outcome: An Analysis Using 16S RRNA Gene Sequencing among IVF Patients, and Trial Therapeutic Intervention for Dysbiotic Endometrium. Reprod. Med. Biol..

[97] Moore D.E., Soules M.R., Klein N.A., Fujimoto V.Y., Agnew K.J., Eschenbach D.A. (2000). Bacteria in the Transfer Catheter Tip Influence the Live-Birth Rate after in Vitro Fertilization. Fertil. Steril..

[98] Moreno I., Garcia-Grau I., Bau D., Perez-Villaroya D., Gonzalez-Monfort M., Vilella F., Romero R., Simón C. (2020). The First Glimpse of the Endometrial Microbiota in Early Pregnancy. Am. J. Obstet. Gynecol..

[99] Ness R.B., Hillier S., Richter H.E., Soper D.E., Stamm C., Bass D.C., Sweet R.L., Rice P. (2003). Can Known Risk Factors Explain Racial Differences in the Occurrence of Bacterial Vaginosis?. J. Natl. Med. Assoc..

[100] Zhou X., Brown C.J., Abdo Z., Davis C.C., Hansmann M.A., Joyce P., Foster J.A., Forney L.J. (2007). Differences in the Composition of Vaginal Microbial Communities Found in Healthy Caucasian and Black Women. ISME J..

[101] Molina N.M., Sola-Leyva A., Haahr T., Aghajanova L., Laudanski P., Castilla J.A., Altmäe S. (2021). Analysing Endometrial Microbiome: Methodological Considerations and Recommendations for Good Practice. Hum. Reprod..

[102] Peric A., Weiss J., Vulliemoz N., Baud D., Stojanov M. (2019). Bacterial Colonization of the Female Upper Genital Tract. Int. J. Mol. Sci..

[103] Liu Y., Wong K.K.-W., Ko E.Y.-L., Chen X., Huang J., Tsui S.K.-W., Li T.C., Chim S.S.-C. (2018). Systematic Comparison of Bacterial Colonization of Endometrial Tissue and Fluid Samples in Recurrent Miscarriage Patients: Implications for Future Endometrial Microbiome Studies. Clin. Chem..

[104] Salter S.J., Cox M.J., Turek E.M., Calus S.T., Cookson W.O., Moffatt M.F., Turner P., Parkhill J., Loman N.J., Walker A.W. (2014). Reagent and Laboratory Contamination Can Critically Impact Sequence-Based Microbiome Analyses. BMC Biol..

[105] Chakravorty S., Helb D., Burday M., Connell N., Alland D. (2007). A Detailed Analysis of 16S Ribosomal RNA Gene Segments for the Diagnosis of Pathogenic Bacteria. J. Microbiol. Methods.

[106] Graspeuntner S., Loeper N., Künzel S., Baines J.F., Rupp J. (2018). Selection of Validated Hypervariable Regions Is Crucial in 16S-Based Microbiota Studies of the Female Genital Tract. Sci. Rep..

